# Impact Resistance of 3D-Printed Continuous Hybrid Fiber-Reinforced Composites

**DOI:** 10.3390/polym15214209

**Published:** 2023-10-24

**Authors:** Ali Akmal Zia, Xiaoyong Tian, Muhammad Jawad Ahmad, Zhou Tao, Luo Meng, Jin Zhou, Daokang Zhang, Wenxin Zhang, Jiangwei Qi, Dichen Li

**Affiliations:** 1 State Key Laboratory for Manufacturing System Engineering, Xi’an Jiaotong University, Xian 710049, China; ziaaliakmal@stu.xjtu.edu.cn (A.A.Z.); jin.zhou@xjtu.edu.cn (J.Z.); 13679127667@163.com (D.Z.); runzewenxin@163.com (W.Z.); jiangweiqi@stu.xjtu.edu.cn (J.Q.); dcli@mail.xjtu.edu.cn (D.L.); 2 Shenzhen 3D Printing Manufacturing Innovation Center, Shenzhen Xietongchuangxin High Tech Development Co., Ltd., No. 19 Lanjin 4th Road, Pingshan District, Shenzhen 511400, China

**Keywords:** 3D printing, material extrusion (MEX), hybrid fiber (HF), composite materials, impact resistance

## Abstract

Improving the resilience of 3D-printed composites through material extrusion technology (MEX) is an ongoing challenge in order to meet the rigorous requirements of critical applications. The primary objective of this research was to enhance the impact resistance of 3D-printed composites by incorporating continuous hybrid fibers. Herein, continuous virgin carbon (1k) and Kevlar (130D and 200D) fibers were used with different weight and volume fractions as reinforcing fibers to produce hybrid and non-hybrid composites for impact resistance testing to obtain energy absorption with different impact energies: 20 J, 30 J, 40 J, and 50 J. Moreover, 0°/90° fiber orientations were used. Hybrid composites with combinations of PLA + CF + 130D KF and PLA + CF + 200D KF showed higher impact resistance, less damaged areas (71.45% to 90.486%), and higher energy absorption (5.52–11.64% more) behaviors compared to PLA + CF non-hybrids. CT scan images provided strong evidence to resist the fracture and breakage patterns, because the stiffness and elongation properties of the fibers acted together in the hybrids specimens. Furthermore, positive hybrid effects of the PLA + CF + KF hybrids also showed an ideal match of toughness and flexibility in order to resist the impacts. In the future, these hybrids will have the potential to replace the single type of composites in the fields of aerospace and automobiles.

## 1. Introduction

Hybrid composites offer a wide range of advantages, including outstanding fatigue and corrosion resistance, as well as impact resistance. By combining two or more lignocellulosic fibers into a single matrix, fiber-reinforced hybrid composites can be created [1,2,3]. The main benefit of using a hybrid composite is that any fiber lacking certain properties can be compensated for by the other fibers [4,5]. Additionally, by carefully considering the material design, a balance between the cost and performance of hybrid composites can be achieved [6]. While the cost and waste of the composites can also be reduced by recycling the fibers [7,8,9], there are various conventional production processes for producing hybrid composite parts. The hand lay-up method, the simplest and oldest technique for creating composites, has great repeatability and quick cycle times [10,11,12]. Compression molding has been the most widely used process for producing industrial composite components [4]. Fiber and matrix debonding (fiber pull-out), delamination, and fiber breakage are three possible pathways of energy absorption and dissipation after impact for fiber-reinforced composites. These mechanisms can reduce the load carrying capability of the composite structures [13,14,15]. Although, in structures, fiber composites have good corrosion resistance, high rigidity, and low weight as positives, they have poor impact resistance. This aspect necessitates additional study to improve the impact resistance of structural textile composites by fusing them with some impact-resistant material while maintaining their exceptional mechanical qualities [16,17]. Therefore, for a damage-tolerant design, impact resistance plays a very important role for a long life for structures, but there have been few attempts to examine the impact behavior of sandwich constructions under impact loading. On the other hand, it is still needed to investigate the impact resistance and energy absorptions properties of such types of hybrids, as mentioned in [18]. However, creating hybrids at the fiber level is a more straightforward and creative strategy.

With the development of 3D printing technology, continuous fiber-reinforced composite components with complicated structures have been fabricated, which has broken through the structural limitation caused by the conventional fabrication process. Continuous fiber or prepreg can be fed into a specially constructed nozzle during the 3D printing process for CFRPCs, and additional matrix materials can be fed into the nozzle at the same time to achieve coextrusion printing. Three-dimensional printing technology is allowed for the preparation and shaping of composite materials, which makes it possible to fabricate multi-scale composite structures. By using 3D printing, the simplest method for fabricating hybrids is the interlayer configuration, which consists of two different fiber types stacked one on top of the other [19]. Recently, hybrid composites were fabricated by the 3D printing process using hybrid threads (HT), with very promising results. By putting mixed fibers into the printing nozzle, hybrid composites based on the continuous fiber 3D printing process have been created by introducing different types of fibers through the same inlet while a polymer is fed through a separate inlet. These hybrids showed substantially higher bending and tension strengths with absorbed energies than traditional single-type fiber composites [18].

As mentioned in the above studies, various aspects of the hybrid composite structure have already been researched, but many issues still remain unresolved, especially for the impact resistance of 3D-printed hybrid composites by material extruded (MEX). According to a research study, there are still numerous unsolved problems regarding how impact loading affects 3D-printed specimens [20,21]. The study highlighted the significance of printed composites and emphasized the improvement in their mechanical properties. In the case of impact tests, numerical calculations demonstrated that they are comparable to experimental observations [22]. In a different investigation, solid fill, lamina thickness, and specimen orientation were used to assess the impact toughness of onyx specimens. Onyx has been found to be more brittle when compared to nylon due to the presence of chopped carbon fibers. Additionally, continuous Kevlar fiber was used to strengthen the test specimens in an isotropic and concentric pattern. The amount of energy absorbed increased when a continuous fiber was present [23]. Due to inadequate matrix polymer impregnation, fiber-rich regions have weaker adhesion, an increase in internal porosity of roughly 2%, and a higher chance of crack initiation and propagation. Moreover, microstructures also depend on the nozzle geometry, overlapping between layers and bead deposition [24]. Moreover, H. Dou et al. [25] investigated the drop weight impact response of 3D-printed CFRPC honeycomb structures. In comparison to nonreinforced composites, the continuous carbon fiber significantly increased the honeycomb structure’s impact resistance. The experimental findings were in good agreement with the finite element model. The internal damage to a honeycomb structure caused by an impact was also detected using computed tomography (CT). The CT images revealed that the main structural failure mechanisms were failures at the matrix–fiber interface and the destruction of adjacent printing channels. However, the impact resistance and energy absorption qualities of this type of hybrids by the material extrusion (MEX) 3D printing process have not been reported yet.

In the present research, in order to investigate the impact resistance of 3D-printed hybrid composites created by material extrusion (MEX), 20 J, 30 J, 40 J, and 50 J of impact energies were used on specimens by using the drop hammer impact test. Energy absorption and hybrid effect behaviors were examined by combining the results from the indicated hybrids. For this, both hybrid and non-hybrid composites with varying weight content values of fibers (such as carbon and Kevlar (130D and 200D) fibers) were used. CT scan examinations were also conducted to examine the breaking characteristics at the microscopic level.

## 2. Experimental Methodology

### 2.1. Materials and Processing

This study utilized polylatic acid (PLA) from eSUN filament Jushi Group Co., Ltd. (Jiaxing, China), which had a diameter of 1.75 mm, served as the effective matrix. 1k continuous commercial virgin carbon fiber from Tory Corp., (Tokyo, Japan), Dupont Kevlar 130D and Kevlar 200D continuous fibers that were selected as reinforcement materials. 130D and 200D Kevlar were combined with 1k carbon fibers to produce hybrid fibers. All the fiber volume fractions are given in Table 1, which were calculated by using the same method as used elsewhere [18]. As PLA reacts differently in different temperatures [26], to reduce the humidity before use, PLA was kept in a dry atmosphere. The FiberTech 3D printer (Xi’an, China) was used to create hybrid and non-hybrid composites with continuous Kevlar and carbon fibers. Continuous Kevlar and carbon fibers entered this FDM machine through the same inlet during operation, while the matrix entered through a different inlet. When the matrix traveled through the heater with the aid of the nozzle, the fibers were impregnated with the melted matrix. The panel was then printed with the composite. In this study, the specimens were printed onto a non-heated platform with hatch spacing of 1 mm, layer thickness of 0.3 mm, nozzle diameter of 1.0 mm, printing speed 200 mm/min, solid infill density, and printing temperature of 210 °C.

### 2.2. Characterization

**Figure 1:**
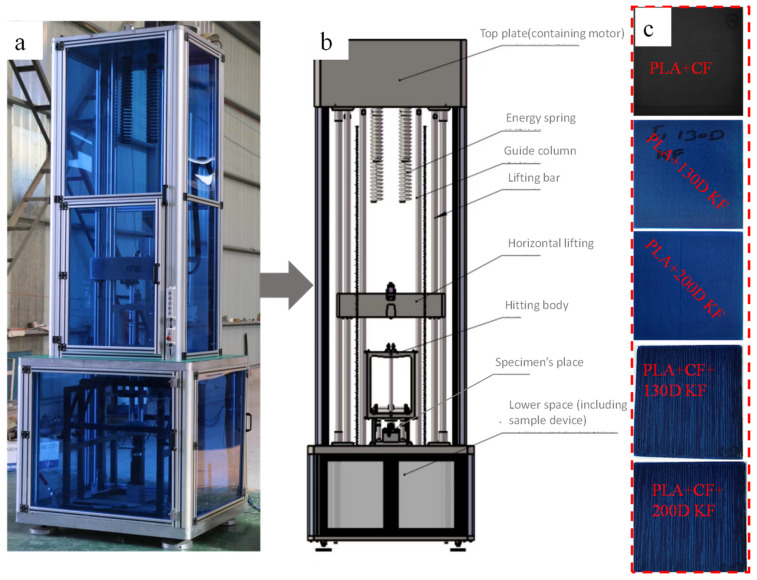
(**a**) XJSTLT-2000 drop weight impactor. (**b**) Schematic working diagram. (**c**) Printed specimens that were tested.

The study investigated the impact resistance of printed composites, both hybrid and non-hybrid, by analyzing factors such as the energy absorption and fracture patterns. Figure 1a,b elucidate the schematic and real diagram of the drop weight impactor (XJSTLT-2000 Drop weight Impactor from Shang Tai Instrument Co., LTD, Guangdong, China), which were used to investigate the above-mentioned performances with 20 J, 30 J, 40 J and 50 J impact energies, a 20 mm impactor diameter, and 6.449 kg impactor weight. 150×100×2.9 dimensions were used to prepare specimens in accordance with ISO 6603 [27]. The Y. Cheetah Micron X-ray 3D imaging system (from YXLON International GmbH, Hamburg, Germany) CT scan machine was used to study the microcracks and fiber breakages. Each specimen contained 9 layers, with 0°/90° printing orientations. All tests were carried out at room temperature in order to counteract the influence of the temperature, which has a considerable impact on the mechanical properties of composites. For one type of test, a total of five different hybrid and non-hybrid specimen types were prepared. Additionally, each type had three samples, allowing us to average the outcomes. The damage areas of the hybrids were compared with PLA + CF composites, because in aerospace and automobiles, mostly carbon fibers are used.

## 3. Results and Discussion

### 3.1. Loading Response

**Figure 2:**
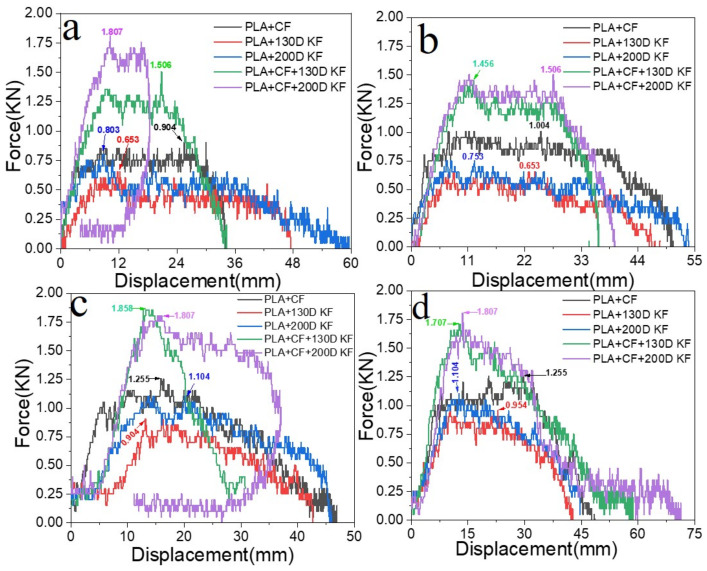
Impact loading reactions on different energy levels: (**a**) 20 J, (**b**) 30 J, (**c**) 40 J, and (**d**) 50 J.

Figure 2a–d depict the loading reactions of the composite specimens that were affected by the impactor. Figure 2a shows the impact loading response of the hybrids and non-hybrids with 20 J of impact energy.

The impact load of PLA + CF + 130D KF was 1.506 kN, which was 1.66 times more than PLA + CF, while PLA + CF + 200D KF resisted the impact load until 1.807 kN, which was two times more than PLA + CF. With 30 J of impact, this difference compared to PLA + CF was 1.45 times and 1.5 times more, respectively, shown in Figure 2b. Furthermore, the impact loads faced by PLA + CF + 130D KF and PLA + CF + 200D KF were 1.48 times and 1.43 times, respectively, higher than PLA + CF at 40 J of impact energy, which is elucidated in Figure 2c. When the specimens were subjected to 50 J of impact energy, illustrated in Figure 2d, again, the impact resistance of the hybrids was 1.36 and 1.43 times higher than non-hybrid PLA + CF. The higher impact loading of the hybrids with less displacement shows that the hybrids are stiffer and stronger than the non-hybrids. In other words, the impact resistance of the hybrids was better than all the non-hybrids. Carbon fibers proved to be a viable fiber for improving their performance [28]; herein, the non-hybrid PLA + CF specimens showed high-impact loads compared to the other non-hybrids, which is why the impact strength of the hybrids was discussed according to the PLA + CF composites.

### 3.2. Material Damage and Impact Resistance

For all the hybrids and non-hybrids, the impact damage was different, and it became more visible with the increase in impact energy from 20 J to 50 J. The specimens right for printing are provided in Figure 1c.

**Figure 3:**
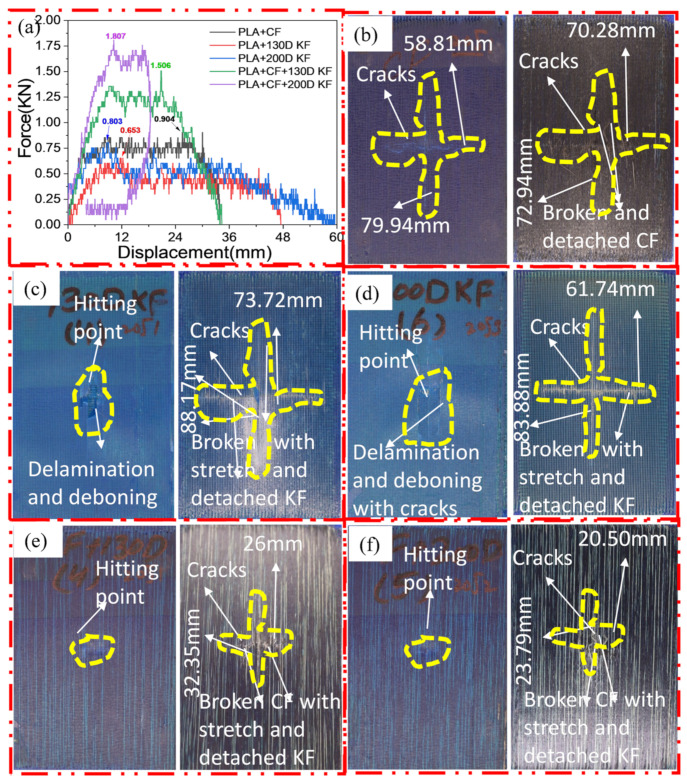
Material damage and impact resistance with 20 J. (**a**) Impact loading reaction. (**b**) Front and back sides of PLA + CF. (**c**) Front and back sides of PLA + 130D KF. (**d**) Front and back sides of PLA + 200D KF. (**e**) Front and back sides of PLA + CF + 130D KF. (**f**) Front and back sides of PLA + CF + 200D KF.

Figure 3a–f shows the damage and impact resistance of the hybrid and non-hybrid composites with 20 J of impact energy. In Figure 3b, cracks are visible in both the front and back sides; in the Kevlar-containing specimens, the delamination was obvious, with damage on the hitting side and back side, showing that the fibers were broken with the stretch, as shown in Figure 3c,d, while, in the hybrids, no damage could be found, just slight indentation marks apparent on the hitting side of the specimens. Moreover, the damage on the back sides the of the PLA + CF + 130D KF hybrids was 83.592% and PLA + CF + 200D KF 90.486% less than the PLA + CF composites. The impact resistive force of these hybrids was higher than the non-hybrids, with minimum damage, which is evidence that the hybrids are more impact resistive than non-hybrids, as shown in Figure 3a,e,f. Further values are listed in Table 2.

**Figure 4:**
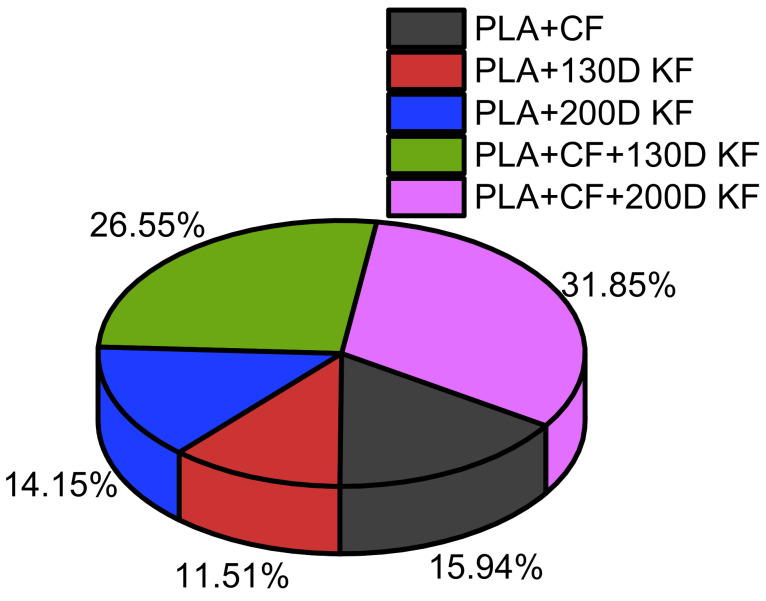
Overall comparison of the resistive forces among the hybrid and non-hybrid composites at 20 J of impact energy.

Considering the sum of all resistive forces of the hybrid and non-hybrid composites is 100%, PLA + CF + 200D KF showed the highest values. Moreover, both hybrids were able to occupy 58.4% of the pie chart in Figure 4, which suggests that the impact resistance of the ductility and brittleness together are superior compared to the impact-resistive property of the single sort of non-hybrid composites.

**Figure 5:**
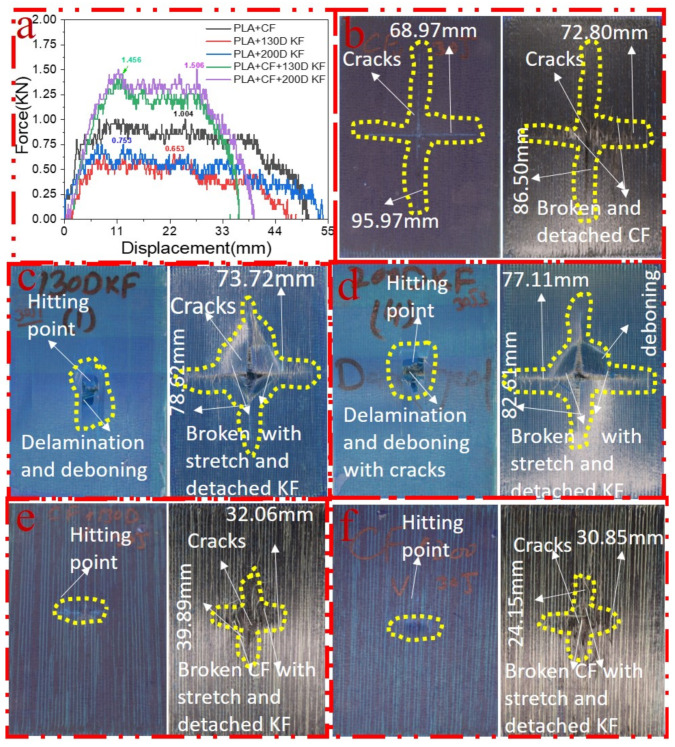
Material damage and impact resistance with 30 J. (**a**) Impact loading reaction. (**b**) Front and back sides of PLA + CF. (**c**) Front and back sides of PLA + 130D KF. (**d**) Front and back sides of PLA + 200D KF. (**e**) Front and back sides of PLA + CF + 130D KF. (**f**) Front and back sides of PLA + CF + 200D KF.

Figure 5a–f elucidate the damage and impact resistance of the hybrid and non-hybrid composites with 30 J of impact energy. In Figure 5b, cracks are aggravated and more visible in both the front and back sides; in the Kevlar-containing specimens, the delamination was obvious, with damage on the hitting side and back side showing that the fibers were broken with the stretch, deboning between the layers, and deeper penetration of the impactor, as shown in Figure 5c,d, while, in the hybrids, just slight indentation marks were apparent on the hitting sides of the specimens. Moreover, the damage on the back sides of the PLA + CF + 130D KF and PLA + CF + 200D KF hybrids was almost 79.69% and 88.16% less than PLA + CF non-hybrids, respectively, which was slightly bigger than the hybrids of 20 J due to the increase in impact energy. Again, the impact forces of the PLA + CF + 130D KF and PLA + CF + 200D KF hybrids were higher than the non-hybrids, with minimum damage, which shows their high strength against impacts, as shown in Figure 5a,e,f. All the concerning values of the hybrid and non-hybrids against 30 J of impact energy are listed in Table 3.

**Figure 6:**
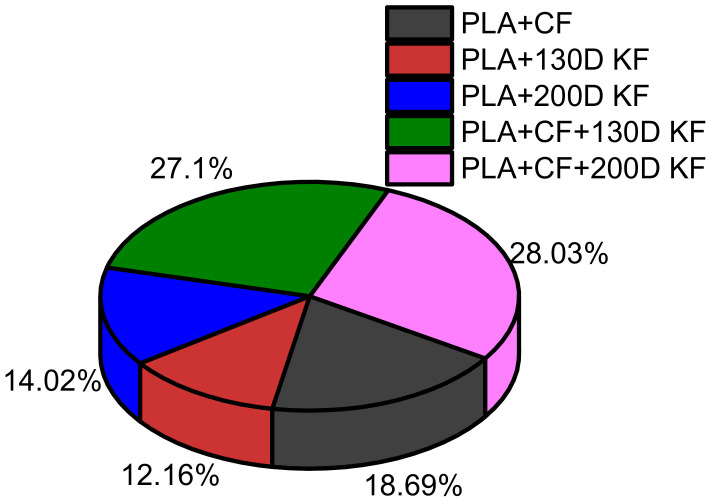
Overall comparison of the resistive forces among the hybrid and non-hybrid composites at 30 J of impact energy.

Figure 6 depicts the dominance of the hybrid composites compared to the non-hybrids in a pie chart with 30 J of impact energy. The PLA + CF + 130D KF was 27.1%, and the PLA + CF + 200D composite was 28.03% of the pie chart. Moreover, the damage areas of these hybrids were also 79.69% and 88.16% less than the PLA + CF composites, as listed in Table 3.

**Figure 7:**
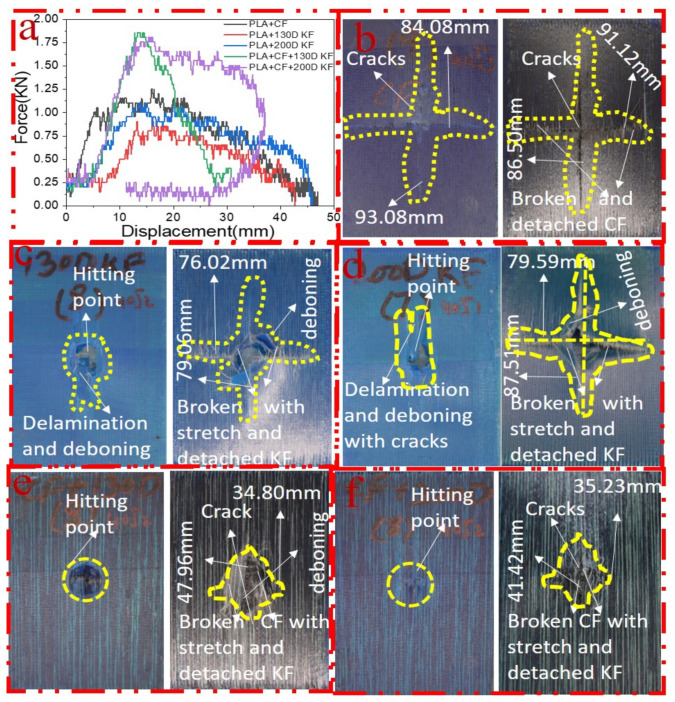
Material damage and impact resistance with 40 J. (**a**) Impact loading reaction. (**b**) Front and back sides of PLA + CF. (**c**) Front and back sides of PLA + 130D KF. (**d**) Front and back sides of PLA + 200D KF. (**e**) Front and back sides of PLA + CF + 130D KF. (**f**) Front and back sides of PLA + CF + 200D KF.

Figure 7a–f elucidate the damage and impact resistance of the hybrid and non-hybrid composites with 30 J of impact energy. In Figure 7b, the cracks are aggravated and more visible in both the front and back sides but without evidence of the impactor’s penetration; in the Kevlar specimens, the delamination was clearer, with damage on the hitting side and back sides showing that the fibers were broken with the stretch, and the deboning between layers were more visible because of the deeper penetration of the impactor, as shown in Figure 7c,d. On the other hand, in the hybrids, damage was visible in the PLA + CF + 130D KF composites, but still, not very deep indentation marks were apparent on the hitting side of the PLA + CF + 200D KF specimens. Moreover, the damage on the back sides of these hybrids was 78.82% and 88.16% less than the PLA + CF non-hybrids, which were slightly bigger than the hybrids of 30 J. Again, the impact resistance forces of the PLA + CF + 130D KF and PLA + CF + 200D KF hybrids were higher than the non-hybrids, with minimum damage, which show their high strength against impacts, as seen in Figure 7a,e,f and Table 4. All the concerning values of the hybrid and non-hybrids against 40 J of impact energy are listed in Table 4.

**Figure 8:**
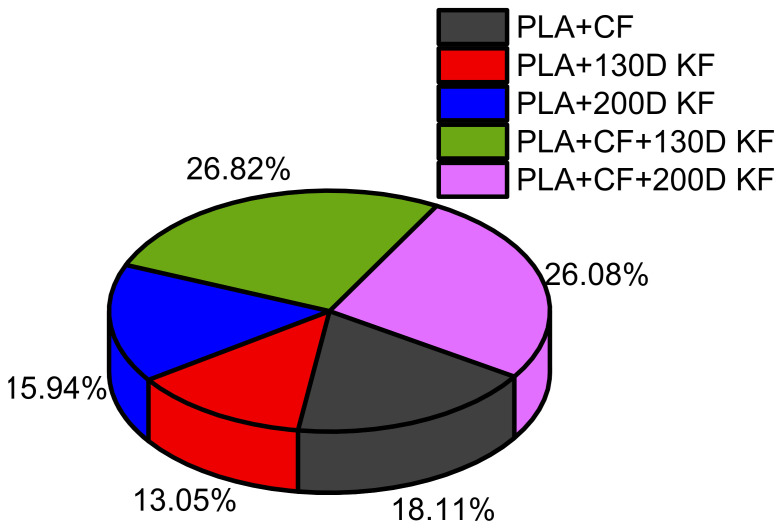
Overall comparison of the resistive forces among the hybrid and non-hybrid composites at 40 J of impact energy.

On the other hand, in Figure 8, the percentages of the resistive impacts of the hybrids were again higher than the non-hybrids, which suggests that the hybrid composites were stable against variations in the impact energies, with minimum damage areas.

**Figure 9:**
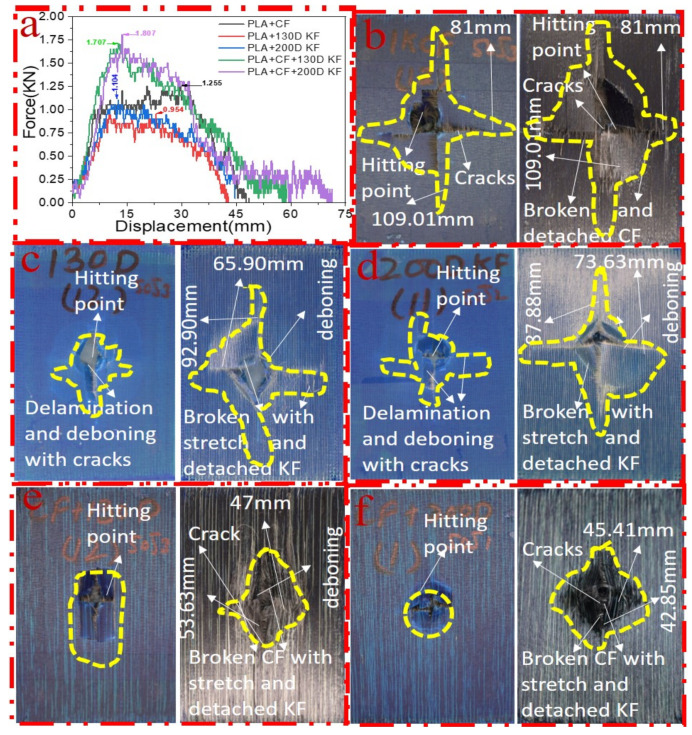
Material damage and impact resistance with 50 J. (**a**) Impact loading reaction. (**b**) Front and back sides of PLA + CF. (**c**) Front and back sides of PLA + 130D KF. (**d**) Front and back sides of PLA + 200D KF. (**e**) Front and back sides of PLA + CF + 130D KF. (**f**) Front and back sides of PLA + CF + 200D KF.

**Figure 10:**
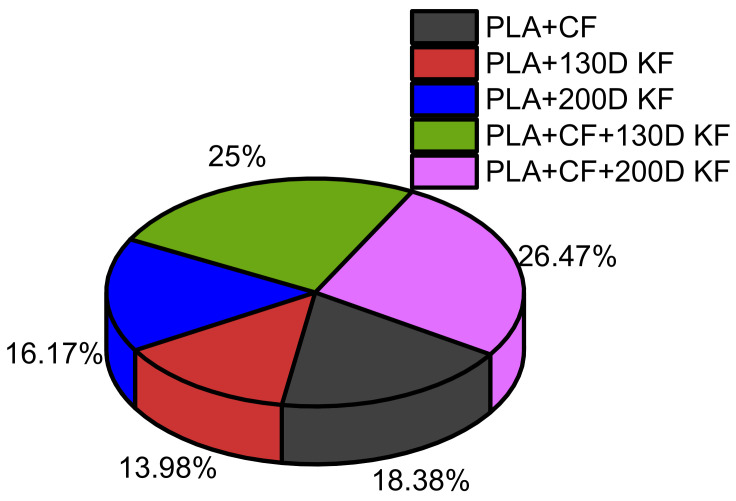
Overall comparison of the resistive forces among the hybrid and non-hybrid composites at 50 J of impact energy.

All the specimens, hybrid and non-hybrids, were damaged with 50 J of impact energy, as shown in Figure 9b–f. According to Figure 9a,b, the PLA + CF specimens were completely damaged with 1.255 kN of impact force because of the brittle nature of the carbon fibers. The damage patterns in PLA + 130D KF and PLA + 200D KF were a little different than the carbon fiber specimens, because in these specimens, the delamination was aggravated on the hitting side and deboning was also more visible with the naked eye, but its impact resistance force was less than the carbon fiber specimens, as depicted in Figure 9a,c,d. On the other hand, in the PLA + CF + 130D KF and PLA + CF + 200D KF hybrids, the impactor penetrated, but some unbroken and stretched fibers were still present on the back sides of the specimens. The damage was clearer, but the propagation of future cracks was hard to see with the naked eye. The impact force was still higher than the non-hybrids. In other words, the hybrids were stronger and more impact cracks-resistive than the non-hybrids, because in hybrids, stiffness and ductility act at the same time to protect the specimen. Figure 10 and Table 5 illustrate the behaviors of he hybrid and non-hybrid composites against 50 J of impact energy. On the other hand, in the literature, single sorts of composites have been used to study the impact resistance of different CF, KF, and GF composites [29]. At room temperature, the failure modes included fiber pull-out and fiber tearing. In the case of fiberglass, the failure mechanism was matrix cracking, while, for HSHT, it involved delamination and matrix cracking. In our case, these phenomena were very less, e.g., no fiber pull-out and very less delamination.

### 3.3. CT Scanning

To see the level of damage caused by impacts with energies of 20 J, 30 J, 40 J, and 50 J, photographs of the damaged hybrid and non-hybrid specimens were captured. Photographs of the front and back were taken. Figure 3, Figure 5, Figure 7, and Figure 9 depict comparable damage patterns that reveal the extent of the damage and the presence of cracks. Furthermore, the specimens were evaluated using a computed tomography (CT) scan to look more closely at the damaged areas near the impact zone and the blind side of the impact damage. Additionally, these scans showed the quality assurance of the specimens, with strong connections of the matrix and fibers. That was why the CT scans were very smooth from the front and back sides.

As can be seen above in Figure 3, Figure 5, Figure 7, and Figure 9, most of the non-hybrid specimens had visible cracks and damage after impact. This is why Table 1 contains the CT scans of only the hybrid fiber composites, and the rest of the CT scans of the non-hybrids are provided in the Supplementary Materials (SM) for further verifications.

According to the CT scan images presented in Table 6, the PLA + CF + 130D KF hybrids had more microcracks than PLA + CF + 200D KF. With 20 J of impact energy, PLA + CF + 130D KF had cracks around the hitting area, and these cracks were aggravated with the increase in impact energy. Moreover, these specimens were punctured with 40 J of impact, and the damage was more severe with 50 J, but there were no further microcracks seen. On the other hand, PLA + CF + 200D KF did not show any microcracks on the front side of the hitting area. The cracks were aggravated more with the increase in impact energy. The aggravation of the cracks in the PLA + CF + 200D KF hybrids was less than PLA + CF + 130D KF, which could be seen with 40 J of impact, where the PLA + CF + 130D KF hybrids were punctured while PLA + CF + 200D KF still only had minor cracks. Furthermore, PLA + CF + 200D KF was fully punctured with 50 J, with less damaged areas, compared to PLA + CF + 130D KF. In result, it would be right to say that the lesser presence of microcracks is also evidence that these hybrids have the ability to resist impacts. Additionally, the rhombus shape of the damage that can be seen in the hybrids and non-hybrids (CT scans are in the Supplementary Materials (SM)) may be because of the 0°/90° printing orientations, so different orientations may have different shapes of damage.

### 3.4. Energy Absorption Behavior

By integrating the areas under the load–displacement curves obtained for the hybrids and non-hybrids in the various test impact energies, the values of energy absorbed were determined.

**Figure 11:**
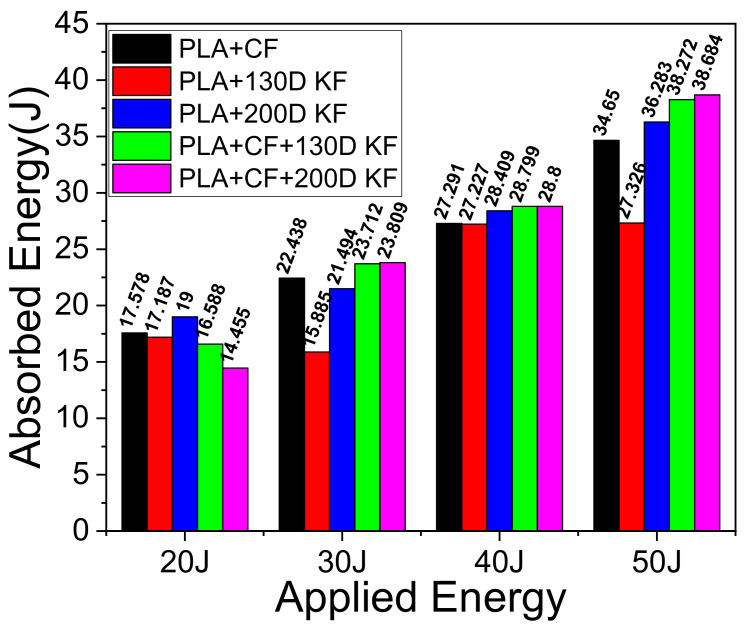
Energy absorption of the hybrid and non-hybrid composites.

Figure 11 and Table 2, Table 3, Table 4 and Table 5 depict the energy absorption behavior of the hybrid and non-hybrid composites. The energy absorption of the hybrids with 20 J was less than the non-hybrids because of high stiffness and impact resistance; the damage was less with a high impact force and less displacement, as shown in Figure 3a,e,f and Table 2. In other words, non-hybrids can absorb the maximum energy with low impact but still cannot resist deformation as much as hybrids. When the specimens were under 30 J of impact, the energy absorption of the hybrids was abruptly higher than all the non-hybrids. Moreover, the energy absorption of the hybrids increased from 5.52% to 11.64% more than PLA + CF with the increase in applied impact energies, as listed in Table 3, Table 4 and Table 5. It was also found that the decrease in impact resistance of the non-hybrids compared to the hybrids was why the damage in the non-hybrids was many times higher than the hybrids, which can be seen in Figure 3, Figure 5, Figure 7, and Figure 9 and in the CT scans as well. By expressing differently, the impactor was stopped by the hybrid specimen’s resistance, yet the specimen was somehow punctured at the impact location due to the high impact energy (40 J and 50 J), while these trends were different in the non-hybrids.

### 3.5. Hybrid Effect

One of the best ways to improve composites’ ability to absorb energy and resist penetration is through hybridization. Here, hybrid effects of the drop hammer impact test specimens stated above are computed as follows:

Using the rule of mixture (*ROM*), the hybrid effect of the printed composites was examined to evaluate the variations in absorbed energy [30,31] using Equation (1) and the ensuing hybrid effect ($h_{e}$) (2).

(1) $$E_{ROM}=\frac{1}{3}\left(E_{\left(PLA+CF\right)}+E_{\left(PLA+130D\;KF\right)}+E_{\left(PLA+200D\;KF\right)}\right)\;$$

In this case, *E_ROM_* denotes the energy absorption mixture rule for those other than the hybrid composites. $E_{\left(PLA+CF\right)}$, $E_{\left(PLA+130D\;KF\right)}$ and $E_{\left(PLA+200D\;KF\right)}$ are the absorbed energy values from the non-hybrid composites that are shown in Figure 11 and Table 2, Table 3, Table 4 and Table 5.

(2) $$h_{e}=\frac{E_{h}}{E_{ROM}}-1$$

where *E_h_* denotes the absorbed energy of the hybrid composites, and *h_e_* denotes the hybrid effect. According to Equation (2), the hybrid effect may have either a positive or negative impact.

If “*h_e_* > 0”, this indicated a positive hybrid effect.

If *h_e_* < 0, a negative hybrid effect was seen due to less than zero values.

**Figure 12:**
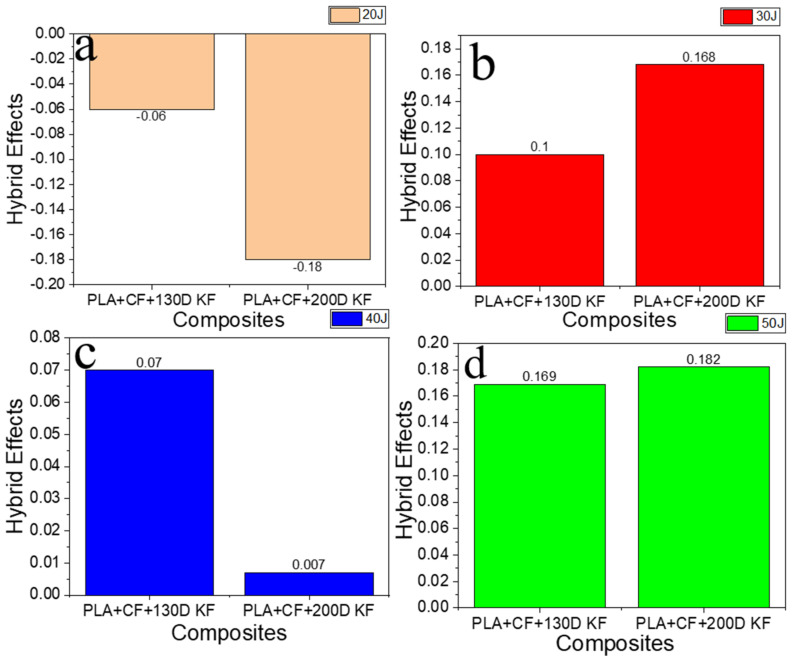
Hybrid effects on different energy levels: (**a**) 20 J, (**b**) 30 J, (**c**) 40 J, and (**d**) 50 J.

Figure 12a–d illustrate the hybrids effects with 20 J, 30 J, 40 J, and 50 J impact energies. The hybrid effects with 20 J were negative because of low energy absorption compared to the non-hybrids, which did not mean that these hybrids were bad with low energy levels, because if comparing these results, combined with the crack resistance and fractures between the hybrid and non-hybrid specimens, then PLA + CF + 130D KF and PLA + CF + 200D KF were two to three times less damaged, as shown in Figure 3, Figure 5, Figure 7, and Figure 9 and Table 2, Table 3, Table 4 and Table 5. Moving forward with higher impact energies, the hybrid effects were positive. Overall, the presented hybrids had good impact resistance and were less damaged, with high-energy absorbers with positive hybrid effects, which are all evidence that hybrid composites are better than non-hybrids in every aspect. Therefore, in the future, the presented composites could be a good option for structural applications in the fields of automobiles and aerospace.

## 4. Conclusions

In this research, the impact resistance of both hybrid and non-hybrid composites was investigated under impact energies ranging from 20 J to 50 J. This evaluation was carried out using the material extrusion (MEX) technique. Most of the comparisons between hybrid and non-hybrids were done with PLA + CF to PLA + CF + 130D KF and PLA + CF + 200D KF because PLA + CF has higher stiffness, and its impact resistive force was also high in all the non-hybrids. Another reason to compare CF composites with hybrids is because of the extensive use of carbon fibers in aerospace and automobiles to analyze the future of the presented hybrids. The following insights could be gleaned from this study:

- Specimens containing only KFs were good with absorbed energy because of their high elongation properties with low stiffness. The damage and cracks were visible in the non-hybrids from 20 J of impact energy, While the hitting sides of the hybrids had just a sign of being hit, and their blind sides had 90.486% less damage than PLA + CF.
- Damages were more obvious with the increase in impact energies. At 30 J of impact energy, the damages in these hybrids were almost 88.16% less than the non-hybrid PLA + CF, which was slightly more than the hybrids of 20 J. Moreover, the damages of these hybrids against 40 J of impact energy were 81.48% less, which was more than the hybrids of 20 J and 30 J. At 50 J of impact, the non-hybrids were almost destroyed completely, while PLA + CF + 130D KF and PLA + CF + 200D KF were only punctured because of their high perforation resistance, and still, these damages were 71.45% and 78.10% less than PLA + CF, respectively.
- There were no further microcracks found in the computed tomography (CT) scans of the hybrids, which means these specimens were crack-resistive. The energy absorptions were higher, with positive hybrid effects, suggesting its validity for structural applications.
- The breakage patterns in all the specimens were rhombus-shaped, which could be because of the 0°/90° printing orientations. In the future, different printing orientations will be applied to study further breakage patterns.
- In future studies, a system will be implemented to monitor the events of impact tests, as they occur too quickly to be observed with the naked eye. Furthermore, the structural applications of these hybrids will be defined specifically in aerospace and automobiles.

## Figures and Tables

**Table 1:** Volume and weight fractions of the fibers and matrix for the impact test.

Specimens	Matrix Volume Fraction (vol. %)	Matrix Weight Fraction (wt. %)	Fiber Volume Fraction (vol. %)	Fiber Weight Fraction (wt. %)
PLA + 1K CF	88 ± 3	83 ± 5	12 ± 3	17 ± 5
PLA + 130D KF	88 ± 3	83 ± 5	12 ± 3	17 ± 5
PLA + 200D KF	88 ± 3	83 ± 5	12 ± 3	17 ± 5
PLA + 1K CF + 130D KF	88 ± 3	83 ± 5	12 ± 3	17 ± 5

**Table 2:** Impact behaviors of the hybrid and non-hybrid composites with 20 J of impact energy.

Specimens	Puncture Force (kN)	Energy (J)	Type of Deformation	Deformed Area (mm^2^)	Deformed Area % w.r.t PLA + CF	Absorbed Engery % w.r.t PLA + CF
PLA + CF	0.904	17.5078	Ductile with complex tearing	$5126.22\;mm^{2}$	As a reference	As a reference
PLA + 130D KF	0.653	17.187	-	$6499.89\;mm^{2}$	26.796% less	1.8% less
PLA + 200D KF	0.803	19	-	$5178.75\;mm^{2}$	1.02% more	8.52% more
PLA + CF + 130D KF	1.506	16.588	initially brittle with arrested complex structure	841.1 mm^2^	83.592% less	5.25% less
PLA + CF + 200D KF	1.807	14.455	initially brittle with arrested complex structure	487.695 mm^2^	90.486% less	17.43% less

**Table 3:** Impact behaviors of the hybrid and non-hybrid composites with 30 J of impact energy.

Specimens	Puncture Force (kN)	Energy (J)	Type of Deformation	Deformed Area (mm^2^)	Deformed Area % w.r.t PLA + CF	Absorbed Engery % w.r.t PLA + CF
PLA + CF	1.004	22.438	Ductile with complex tearing	$6297.2\;mm^{2}$	As a reference	As a reference
PLA + 130D KF	0.653	15.855	-	$5795.86\;mm^{2}$	7.96% less	29.33% less
PLA + 200D KF	0.753	21.494	-	$6370.05\;mm^{2}$	1.15% more	4.20% less
PLA + CF + 130D KF	1.456	23.712	initially brittle with arrested complex structure	1278.87 mm^2^	79.69% less	5.72% more
PLA + CF + 200D KF	1.506	23.809	initially brittle with arrested complex structure	745.02 mm^2^	88.16% less	6.11% more

**Table 4:** Impact behaviors of the hybrid and non-hybrid composites again 40 J of impact energy.

Specimens	Puncture Force (kN)	Energy (J)	Type of Deformation	Deformed Area (mm^2^)	Deformed Area % w.r.t PLA + 1k CF	Absorbed Engery % w.r.t PLA + CF
PLA + CF	1.255	27.291	Ductile with complex tearing	$7881.88\;mm^{2}$	As a reference	As a reference
PLA + 130D KF	0.904	27.227	-	$6010.14\;mm^{2}$	23.74% less	0.2% less
PLA + 200D KF	1.104	28.409	-	$6964.92\;mm^{2}$	11.63% more	4.09% more
PLA + CF + 130D KF	1.858	28.799	initially brittle with arrested complex structure	1669.0 mm^2^	78.82% less	5.52% more
PLA + CF + 200D KF	1.807	28.8	initially brittle with arrested complex structure	1459.22 mm^2^	81.48% less	5.52% more

**Table 5:** Impact behaviors of the hybrid and non-hybrid composites again 50 J of impact energy.

Specimens	Puncture Force (kN)	Energy (J)	Type of Deformation	Deformed Area (mm^2^)	Deformed Area % w.r.t PLA + 1k CF	Absorbed Engery % w.r.t PLA + CF
PLA + CF	1.255	34.65	Ductile with complex tearing	$8829.81\;mm^{2}$	As a reference	As a reference
PLA + 130D KF	0.954	27.326	-	$6122.11\;mm^{2}$	30.66% less	21.13% less
PLA + 200D KF	1.104	36.283	-	$6470.60\;mm^{2}$	26.71% more	4.71% more
PLA + CF + 130D KF	1.707	38.272	initially brittle with arrested complex structure	2520.61 mm^2^	71.45% less	10.45% more
PLA + CF + 200D KF	1.807	38.684	initially brittle with arrested complex structure	1933.55 mm^2^	78.10% less	11.64% more

**Table 6:** Computed tomography (CT) scans of the hybrid specimens.

PLA + CF + 130D KF			PLA + CF + 130D KF	
Front		Back	Front	Back
20 J	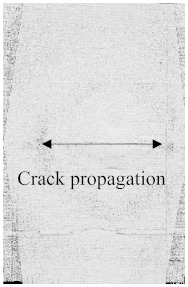	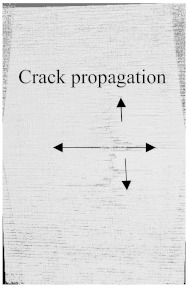	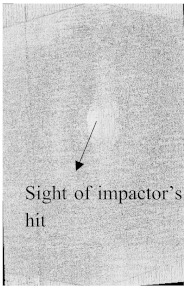	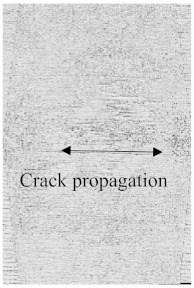
30 J	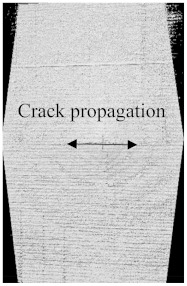	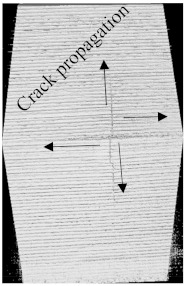	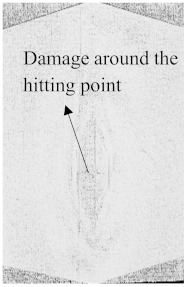	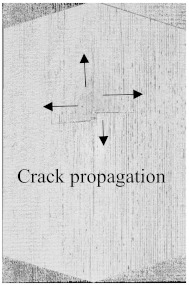
40 J	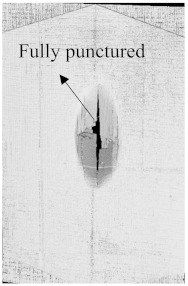	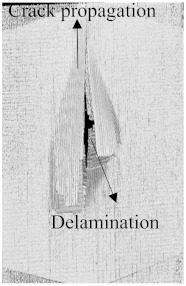	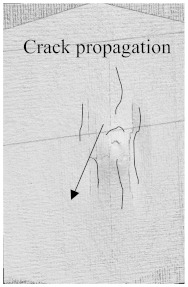	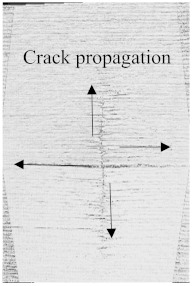
50 J	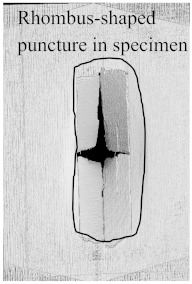	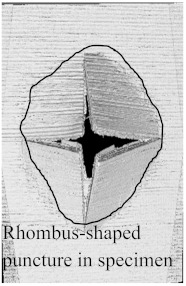	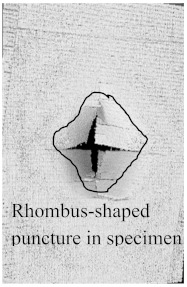	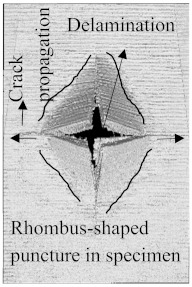

## Data Availability

No new data were created.

## Supplementary Materials

The following supporting information can be downloaded at https://www.mdpi.com/article/10.3390/polym15214209/s1: Figure S1: 20J of impact energy on non-hybrids, 1. hitting point 2. crack propagations 3. Fiber and filament breakage 4. back side damage due to front side hit. 5. layer breakage 6. Delamination; Figure S2: 30J of impact energy on non-hybrids, 1. hitting point 2. crack propagations 3. Fiber and filament breakage 4. back side damage due to front side hit. 5. layer breakage 6. Delamination; Figure S3: 40J of impact energy on non-hybrids, 1. hitting point 2. crack propagations 3. Fiber and filament breakage 4. back side damage due to front side hit. 5. layer breakage 6. Delamination; Figure S4: 50J of impact energy on non-hybrids, 1. hitting point 2. crack propagations 3. Fiber and filament breakage 4. back side damage due to front side hit. 5. layer breakage 6. Delamination.
