# Supplementary material for: Impact Resistance of 3D-Printed Continuous Hybrid Fiber-Reinforced Composites
Source: Polymers (Basel). 2023 Oct 24;15(21):4209. doi: 10.3390/polym15214209 (PMC10648758; doi:10.3390/polym15214209)
Supplement: Supplementary file 1 [file polymers-15-04209-s001.zip › polymers-2658282-supplementary.pdf]

Impact resistance of 3D printed continuous hybrid fiber reinforced composites.

Ali Akmal Zia[1], Xiaoyong Tian[1*], Muhammad Jawad Ahmad[1], Zhou Tao[1], Jin Zhou[1], Daokang Zhang [1], Zhang Wenxin [1], Jiangwei Qi[1], Dichen Li[l].

1. State Key Laboratory for Manufacturing System Engineering, Xi'an Jiaotong University, Xian, Shaanxi,710049, China.

*Correspondence: leoxyt@xjtu.edu.cn;

20J of impact energy on non-hybrids

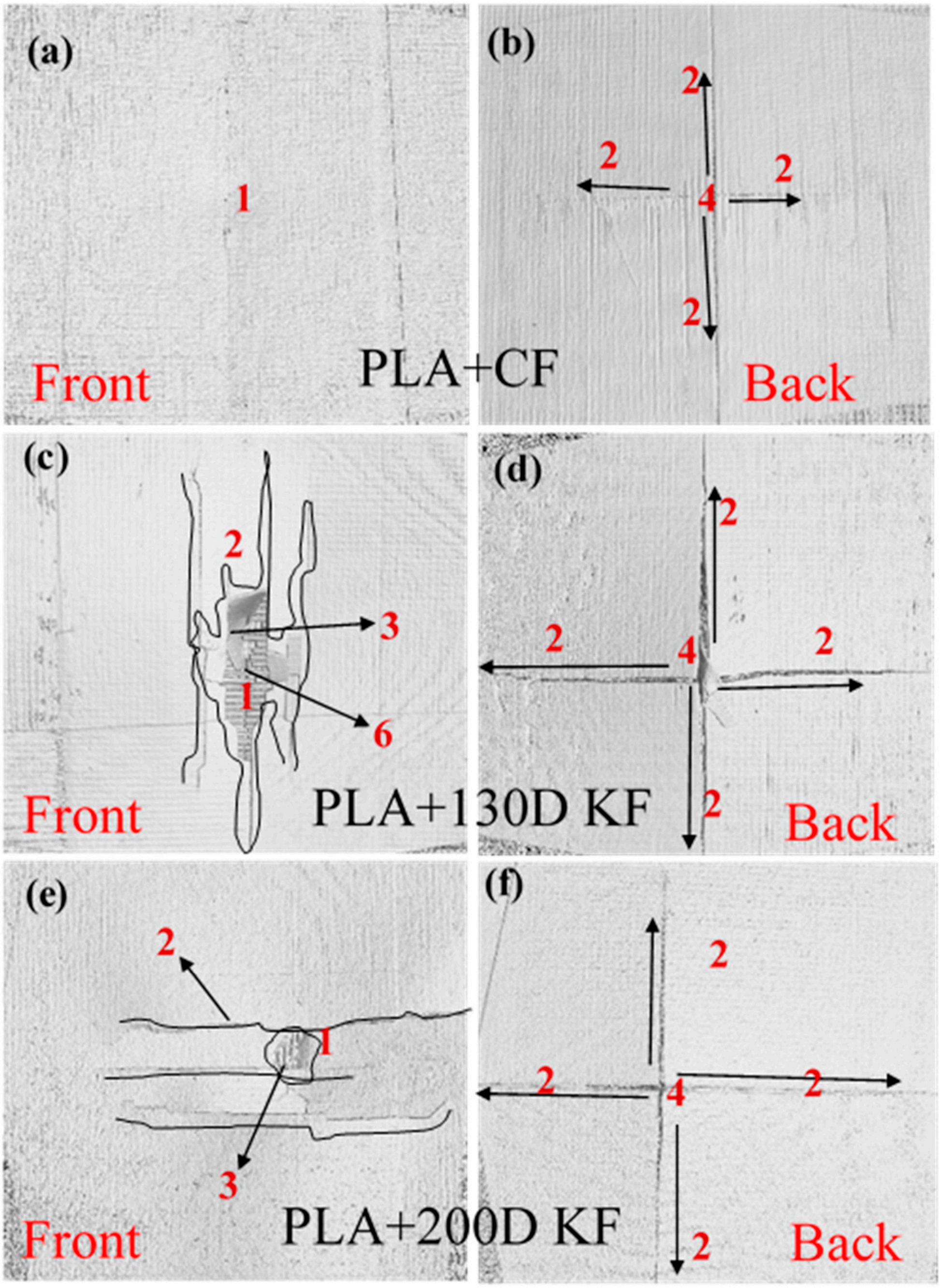

Figure S1. 20J of impact energy on non-hybrids, 1. hitting point 2. crack propagations 3. Fiber and filament breakage 4. back side damage due to front side hit. 5. layer breakage 6. Delamination

30J of impact energy on non-hybrids

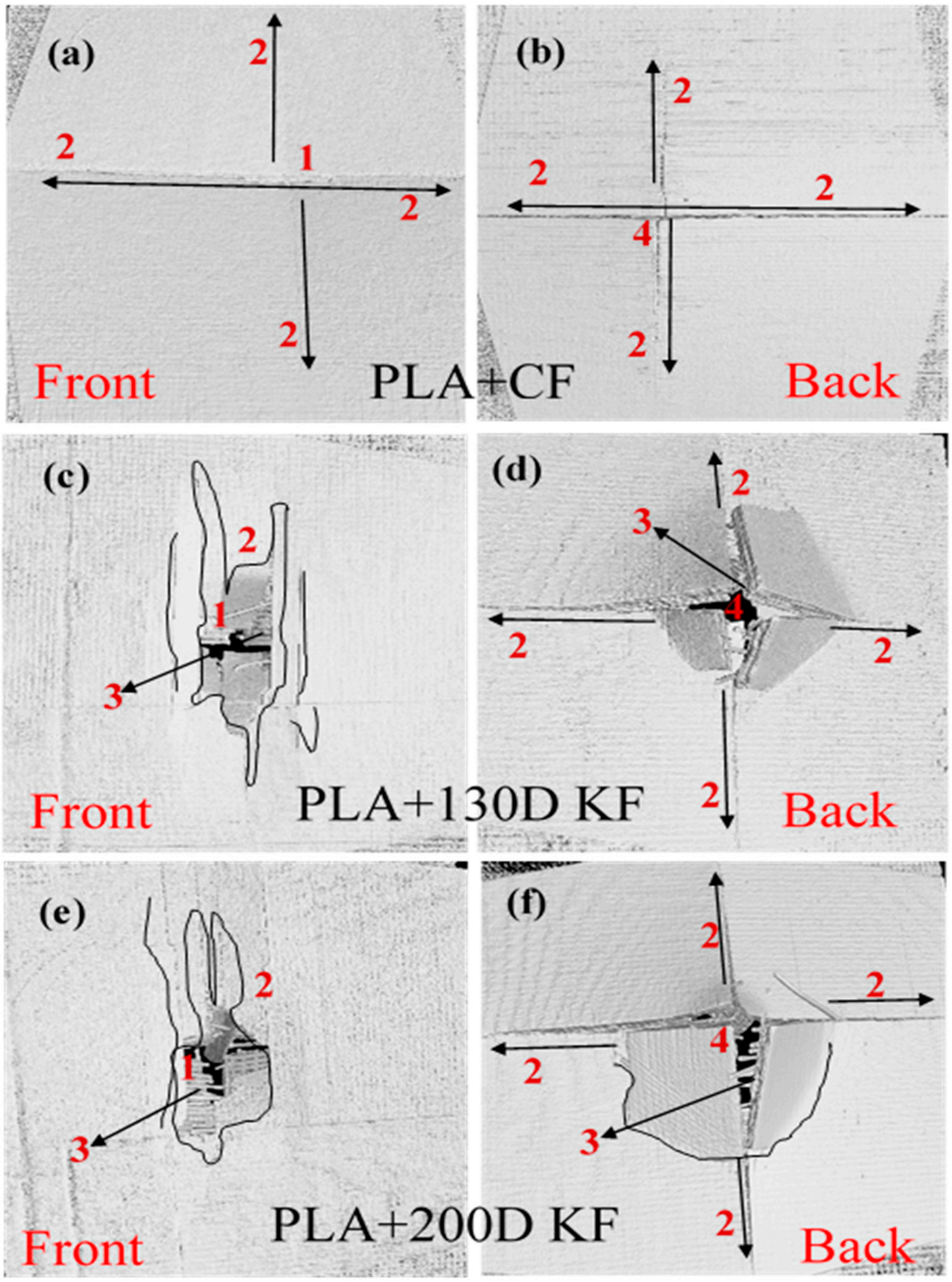

Figure S2. 30J of impact energy on non-hybrids, 1. hitting point 2. crack propagations 3. Fiber and filament breakage 4. back side damage due to front side hit. 5. layer breakage 6. Delamination

40J of impact energy on non-hybrids

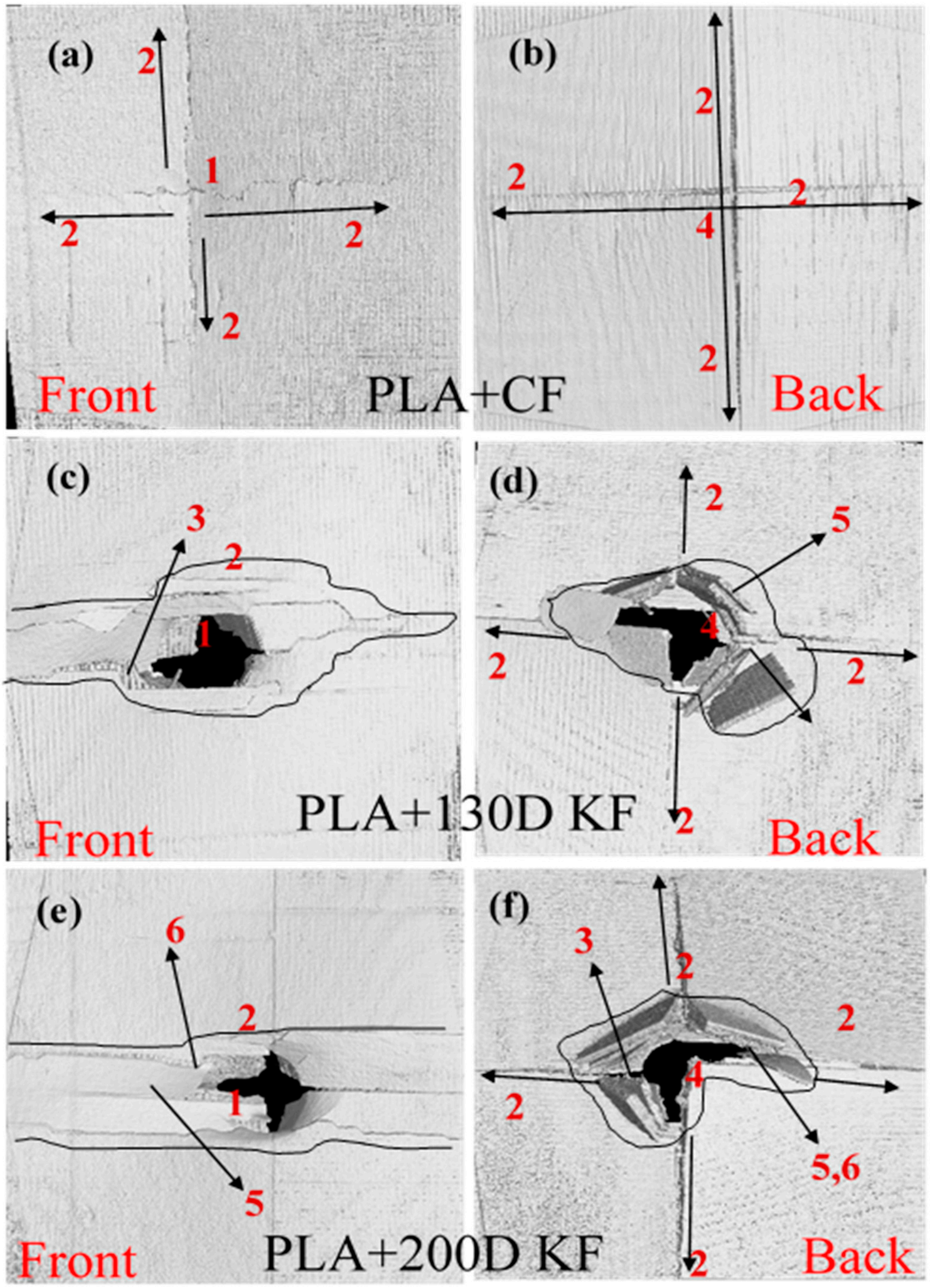

Figure S3. 40J of impact energy on non-hybrids, 1. hitting point 2. crack propagations 3. Fiber and filament breakage 4. back side damage due to front side hit. 5. layer breakage 6. Delamination

50J of impact energy on non-hybrids

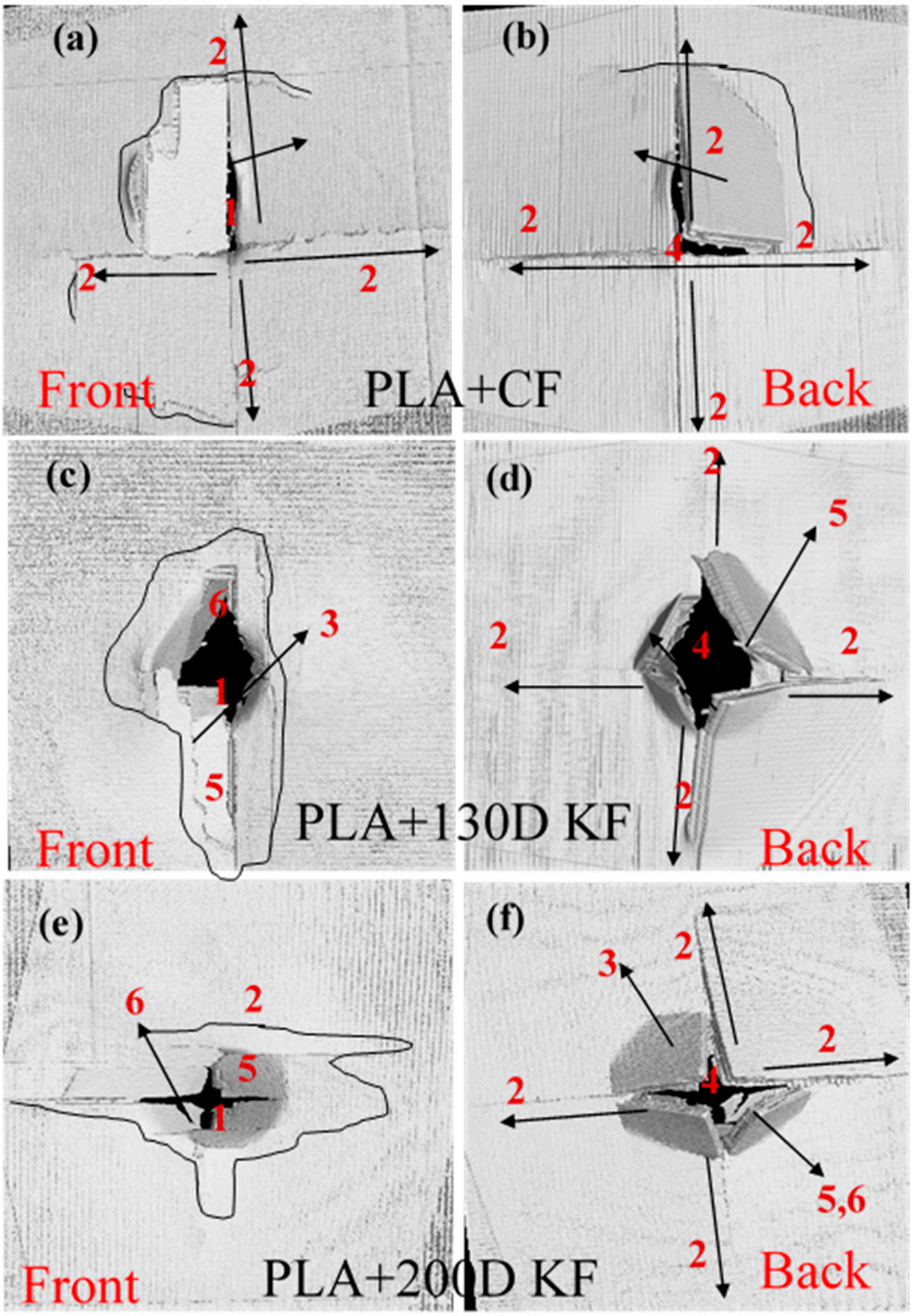

Figure S4. 50J of impact energy on non-hybrids, 1. hitting point 2. crack propagations 3. Fiber and filament breakage 4. back side damage due to front side hit. 5. layer breakage 6. Delamination
